# Evaluation of Light-Activated Provisional Resin Materials for Periodontal Soft Tissue Management

**DOI:** 10.1155/2016/1209705

**Published:** 2016-09-08

**Authors:** Soo-Kyung Jun, Hae-Hyoung Lee, Jung-Hwan Lee

**Affiliations:** ^1^Department of Biomaterials Science, College of Dentistry, Dankook University, Cheonan, Republic of Korea; ^2^Institute of Tissue Regeneration Engineering, Dankook University, Cheonan, Republic of Korea

## Abstract

The purpose of this study was to determine mechanical properties using a compressive test with cylinder specimen (*h* = 6 mm and *ϕ* = 4 mm) as well as cytotoxicity using elutes from disk specimen (*ϕ* = 10 mm and *h* = 2 mm) against human gingival fibroblasts and oral keratinocytes with light-activated provisional resin materials (Revotek LC and Luxatemp Solar) compared to chemically activated counterpart (Snap, Trim II, and Jet). Significantly increased compressive strength (210~280 MPa) was detected in light-activated products compared to chemically activated ones (20~65 MPa, *P* < 0.05) and similar compressive modulus was detected in both types (0.8~1.5 and 0.5~1.3 GPa). Simultaneously, the light-activated products showed less adverse effects on the periodontal soft tissue cells in any polymerization stage compared to the chemically activated products. Particularly, chemically activated products had significantly greater adverse effects during the “polymerizing” phase compared to those that were “already set” (*P* < 0.05), as shown in confocal microscopic images of live and dead cells. In conclusion, light-activated provisional resin materials have better mechanical properties as well as biocompatibility against two tested types of oral cells compared to the chemically activated counterpart, which are considered as more beneficial choice for periodontal soft tissue management.

## 1. Introduction

Many new biomaterials and technologies have been introduced both to replace missing or damaged dental tissues and to promote dental tissue regeneration [1–5]. Among these tissues, periodontal soft tissue has been highlighted for its aesthetic characteristics and inflammation resistance to the oral environment [6, 7]. Therefore, periodontal soft tissue management during and after teeth/implant restoration is essential for esthetic soft tissue contour along with dental restoration, particularly for anterior tooth region.

Provisional restoration is a key step in providing healthy and aesthetically pleasing periodontal soft tissue as well as in maintaining the marginal integrity of prepared teeth/implants and ensuring occlusal function until final prosthodontics are adjusted [8]. Provisional resin materials are widely used for interim restorations due to their ease of handling, suitable mechanical properties, time saving ability, and low cost [9]. Light-curable products have recently been introduced and have beneficial qualities, such as decreasing shrinkage and the time-consuming setting time and increased favorability to patients because of less toxic odor [10].

The clinical use of provisional resin materials is classified into two categories based on how solidly the resins are polymerized from the monomer: (1) traditional, chemically activated materials and (2) recently developed, light-activated materials, including products that are dually activated by chemicals and light. Depending on the how these materials are solidified, their biocompatibility to periodontal tissue and their mechanical properties can differ. Both are crucial for maintaining a natural periodontal tissue appearance in accordance with the tooth/implant contour.

When interim restoration using provisional resin materials is accomplished and positioned around periodontal soft tissue, including the teeth/implant margin and gingival sulcus, toxic components of these materials can adversely affect the tissue via saliva and induce soft tissue management failure [11, 12]. Even worse, particularly in chemically activated materials, “polymerizing” provisional resin materials accelerate adverse effects to periodontal soft tissue because “polymerizing” provisional resin materials increase adverse effects from unreacted monomers or increased concentrations of eluates in the polymerizing phase when they are extracted in the oral cavity [13–15]. Light-activated materials can release fewer cytotoxic components due to their quick setting time (less than 20 s) in the oral cavity and are considered more biocompatible than long-setting, chemically activated materials. In addition, high mechanical properties are positively assumed in light-activated materials due to polymer materials' (UDMA and bis-acryl) natural characteristics, which have better mechanical properties than do PEMA and PMMA [16]. When provision resin materials are broken due to biting force in the oral cavity, the broken pieces can damage periodontal soft tissue and periodontal soft management cannot be successfully performed because of the loss of the provisional restoration [17].

Therefore, in this experiment, we performed cytotoxicity tests to determine the effects on periodontal soft tissue consisting of human oral keratinocytes and gingival fibroblasts during the initial, intermediate, and final polymerizing stages of the provisional resin materials. In addition, as representative mechanical properties, compressive strength and modulus were measured. The first null hypothesis was that the mechanical properties of light-activated provisional resin materials would not differ from those of their chemically activated counterparts. In terms of the biocompatibility, the second null hypothesis was that the cytotoxic effects from light-activated provisional resin materials of extracts on human oral cells would not differ from those of their chemically activated counterparts. The third null hypothesis was that cytotoxic effects to human oral cells would not differ between “polymerizing” and “already set” provisional resin materials.

## 2. Materials and Methods

### 2.1. Provisional Resin Materials

The most commonly used provisional resin materials were chosen for this experiment and included chemically activated polyethyl methacrylate (PEMA) (SN: Snap, Parkell Inc., and TR: Trim, Bosworth), chemically activated polymethyl methacrylate (PMMA) (JE: Jet, Lang Dental), light-activated urethane dimethacrylate (UDMA) and bis-acryl (RL; Revotek LC, GC America), and LS (Luxatemp Solar, DMG). The study involved testing the materials, which are summarized in Table 1.

### 2.2. Preparation of Materials

Three types of chemically activated provisional resin materials (SN, TR, and JE) and two types of light-activated materials (RL and LS) were handled according to the manufacturers' instructions. For in vitro testing, when the chemically activated provisional resin materials reached the early “dough” stage, they were packed into a Teflon mold (*ϕ* = 10 mm, *h* = 2 mm) and covered with a glass plate. Specimens were removed from the molds at three different setting times (25% set, 50% set, and 100% set), as recommended in the manufacturers' instructions, and were immediately placed in distilled water (DW). After the light-activated materials (RL and LS) had been placed on the Teflon mold, the RL specimen was either left unpolymerized or polymerized by means of an LED curing light gun (Litex 695, Dentamerica Industry) for 10 or 20 s. LS specimens were mixed using an automixing gun and placed in the mold for 1 minute or 4 minutes before being placed in DW. For fully polymerized specimens, 10 s light curing using an LED curing light gun (Litex 695) was performed after 4 minutes of chemical activation according to the manufacturer's recommendations. The experimental conditions are detailed in Table 2.

**Figure 1:**
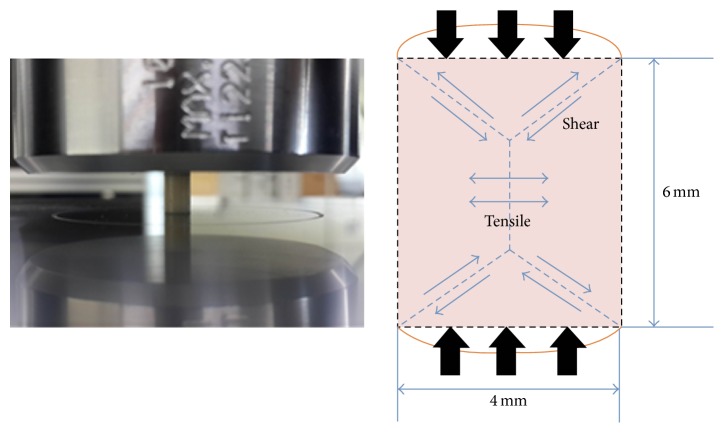
Photograph and schematic diagram of specimens for compressive testing.

For compressive testing, specimens were produced using a stainless steel mold (*h* = 6 mm and *ϕ* = 4 mm, Figure 1). When the chemically activated materials reached the dough stage, they were packed into the mold and covered by celluloid strip and a glass plate, over which an equal amount of pressure was applied during complete polymerization. The RL specimens were compacted into the mold using a resin instrument. Celluloid strips were placed on top of the mold, pressed flat using a glass plate, and allowed to cure for 20 s from both the bottom and the top according to the manufacturer's specifications. The LS specimens were mixed using an automixing tip, placed into the mold, initially cured for 4 minutes, and then light-cured for complete polymerization for 20 s from both the bottom and the top. All materials were further allowed to set even after the final setting time in a static loading device to which 5 kg of load was applied for 3 minutes for complete polymerization. The mold was carefully split, and the specimens were removed and examined to exclude those with porosity or defects. The excess was removed using a diamond bur and specimens were wet polished up to 1200-grit SiC paper. Specimens were immediately immersed in DW and stored in an incubator for 24 hours at 37°C before mechanical testing.

### 2.3. Collection of Resin Extract

Extracts were obtained at a ratio of 3 cm^2^/mL following the recommendations of ISO 10993-12 [18]. Because the surface of the specimens was 2.2 cm^2^, they were incubated in 0.73 mL of medium. Extracts were collected for 24 hours at 37°C in a shaking incubator (120 rpm). All samples were checked for expiration dates and stored according to the manufacturers' recommended conditions throughout the experiment. All procedures were performed on a clean bench to prevent contamination of the specimens.

### 2.4. Culture of Oral Cells

Immortalized human oral keratinocytes (IHOK) and immortalized human normal gingival fibroblasts (hNOF) were used to mimic cytotoxicity to oral mucosal tissue [19, 20]. IHOK and hNOF were generously provided by Professor Dolphine Oda from the Department of Oral Biology from the University of Washington (Seattle, WA) and Professor Jin Kim from the Department of Oral Pathology and Oral Cancer Research Institute from Yonsei University College of Dentistry (Seoul, Korea), respectively. The IHOK and hNOF were cultured in a DMEM/F-12 3 : 1 mixture (Welgene, Daegu, Korea) supplemented with 10% fetal bovine serum (Gibco) and 1% penicillin/streptomycin (Invitrogen) in a humidified atmosphere of 5% CO_2_ at 37°C [20, 21].

### 2.5. Cytotoxicity Tests and Cell Viability

Cytotoxicity tests were performed according to ISO 10093-5 [22]. Briefly, 1 × 10^4^ cells/well were cultured in a 96-well plate (SPL Life Sciences) with 100 *μ*L of supplemented medium for 24 hours. After being washed with phosphate buffered saline (PBS), the cells were cocultured with 50 *μ*L of 2x supplemented medium and 50 *μ*L of extract or a serially diluted extract by DW for another 24 hours. The percentages of the final concentrations of extract in the culture media were 50% and 25%; 50 *μ*L of DW with 50 *μ*L 2x supplemented medium was used as the control.

Cell viability was measured using an MTS assay (CellTiter 96 Aqueous One Solution Cell Proliferation Assay, Promega) according to the manufacturer's protocol, and the results were expressed as the percentage of optical density of each test group compared with each control group (*n* = 6). Optical absorbance was read at 490 nm using a microplate reader (SpectraMax M2e, Molecular Devices). Confocal microscopic images of live and dead cells were obtained to confirm the cytotoxicity results. After the cells were washed with PBS and stained with calcein AM (0.5 *μ*M) and ethidium homodimer-1 (4 *μ*M) (Molecular Probes, Eugene) for 30 minutes, they were examined under a confocal laser microscope (LSM 700, Carl Zeiss). Green fluorescence was observed from the live cells and bright red fluorescence from the dead cells. All analyses were independently performed in triplicate, and the representative means ± standard deviations or images were shown.

### 2.6. Measurement of Compressive Strength and Modulus

The specimens (*h* = 6 mm and *ϕ* = 4 mm), which were fully polymerized, were positioned on the universal testing machine (Instron 5966, MA, USA) for compressive testing. The cross-head speed of this machine was set at 1.0 mm/min using the ±10 kN of load cell. Compressive strength was calculated by dividing the maximum load, expressed in kN, by the original cross section area, expressed in m^2^ of a specimen in a compression test (N/m^2^). The compressive modulus was calculated according to the equation described in other studies [23].

### 2.7. Statistical Analysis

Data were analyzed by a one-way ANOVA with a Duncan post hoc test. Statistical significance was set at 0.05. All statistical analyses were performed using the SPSS version 21.0 software program (SPSS Inc., IL, Chicago, USA).

## 3. Results

### 3.1. Compressive Test

**Figure 2:**
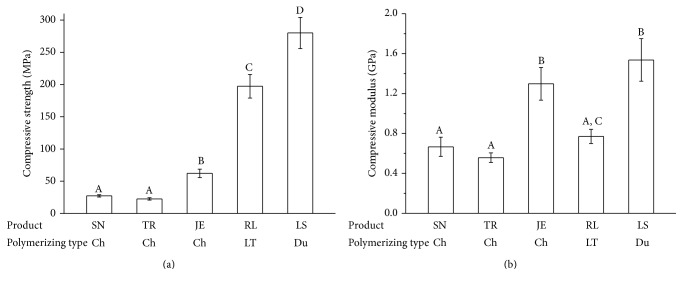
Compressive strength (a) and compressive modulus (b) of provisional restoration materials. Different letters indicate significant differences among the extract conditions (*P* < 0.05).

Figure 2 shows the results of the compressive strength and modulus testing. As seen by the mean compressive strength values of the light-cured groups, LS showed the highest value (280.0 MPa), and RL showed the second highest value (197.4 MPa). TR showed the lowest value (22.6 MPa) among the tested products. SN and JE showed significantly lower compressive strength (27.4 and 62.1 MPa, resp.) compared to the light-activated products (RL and LS). In terms of the compressive modulus, two chemically activated products (SN and TR) and one light-activated product (RL) showed lower values less than 0.8 GPa, while JE and LS had higher compressive modulus values (1.3~1.5 GPa) than did the other specimens (*P* < 0.05).

### 3.2. Cytotoxicity

**Figure 3:**
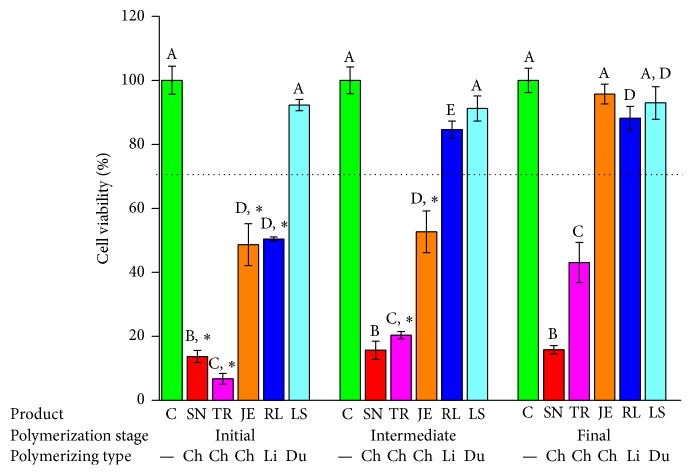
Cell viability results for provisional resin materials according to the extract condition (initial or intermediate polymerizing and already polymerized) and 50% extract concentration in coculture with hNOF. Different letters indicate significant differences in the same extract condition (*P* < 0.05). An asterisk indicates a significant difference compared to the fully polymerized group. A dotted line represents 70% of cell viability.

**Figure 4 fig4:**
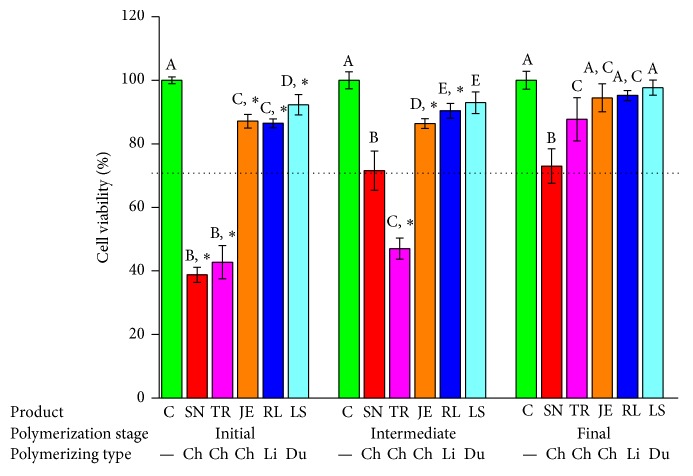
Cell viability results for provisional resin materials according to the extract condition (initial or intermediate polymerizing and already polymerized) and 25% extract concentration in coculture with hNOF. Different letters indicate significant differences in the same extract condition (*P* < 0.05). An asterisk indicates a significant difference compared to the fully polymerized group. A dotted line represents 70% of cell viability.

**Figure 5 fig5:**
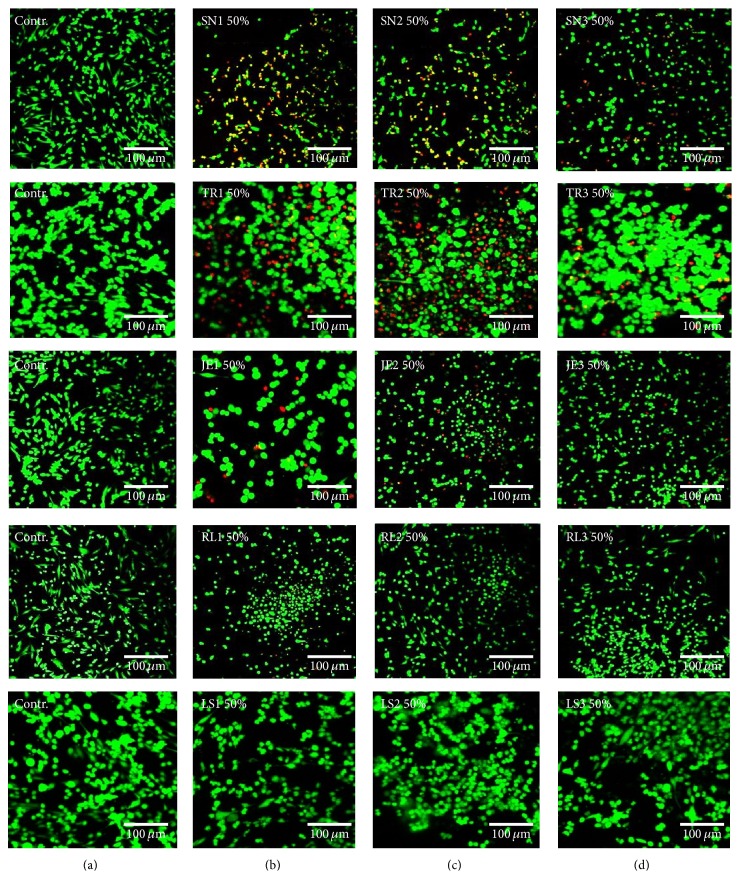
Live (green) and dead cells (red) were observed on confocal microscopy images in hNOF. Images of live and dead cells for media supplemented with 50% distilled water (DW) as control (a). (b) 50% initial extract. (c) 50% intermediate extract. (d) 50% extract from fully polymerized material.

**Figure 6 fig6:**
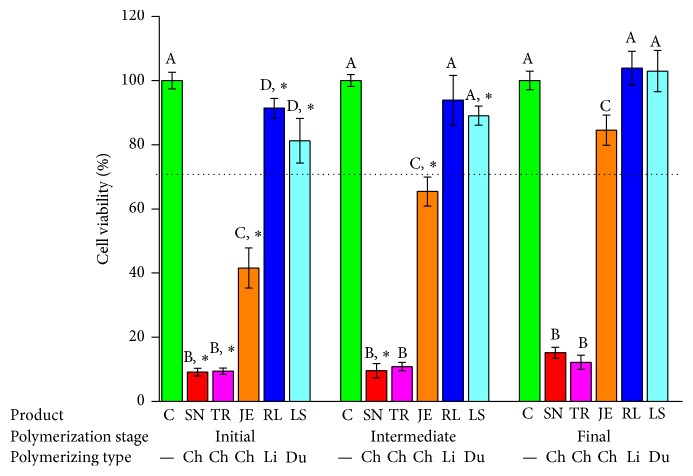
Cell viability results for provisional resin materials according to the extract condition (initial or intermediate polymerizing and already polymerized) and 50% extract concentration in coculture with IHOK. Different letters indicate significant differences in the same extract condition (*P* < 0.05). An asterisk indicates a significant difference compared to the fully polymerized group. A dotted line represents 70% of cell viability.

**Figure 7 fig7:**
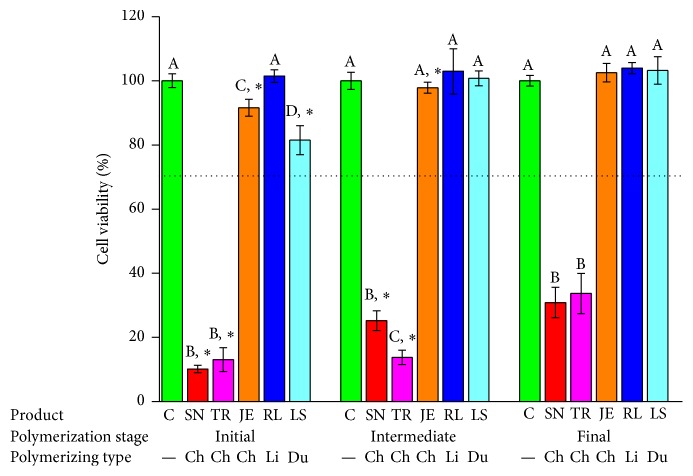
Cell viability results for provisional resin materials according to the extract condition (initial or intermediate polymerizing and already polymerized) and 25% extract concentration in coculture with IHOK. Different letters indicate significant differences in the same extract condition (*P* < 0.05). An asterisk indicates a significant difference compared to the fully polymerized group. A dotted line represents 70% of cell viability.

**Figure 8:**
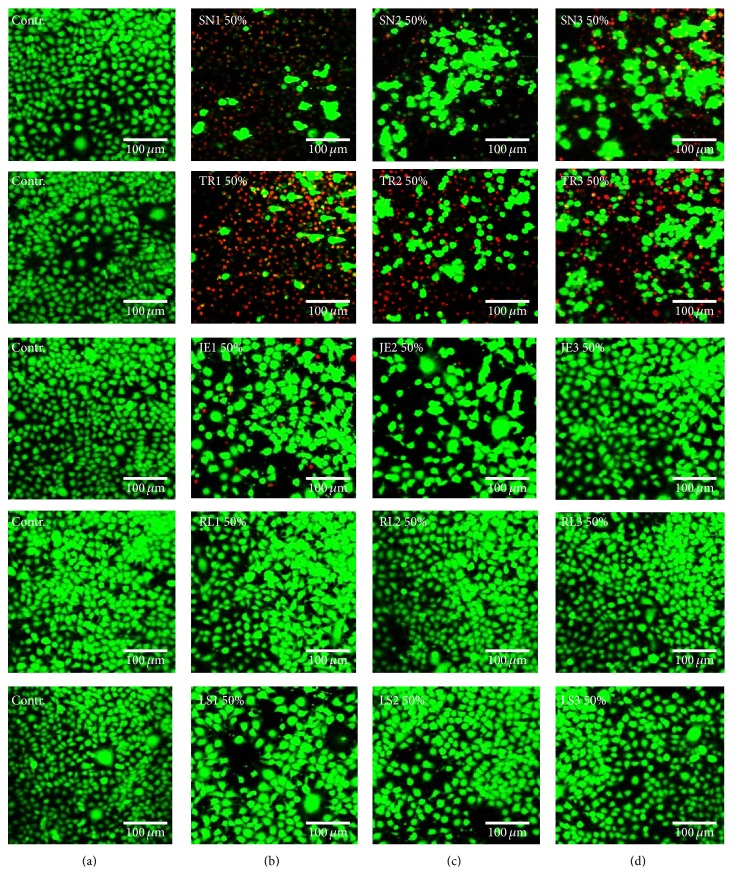
Live (green) and dead cells (red) were observed on confocal microscopy images in IHOK. Images of live and dead cells for media supplemented with 50% distilled water (DW) as control (a). (b) 50% initial extract. (c) 50% intermediate extract. (d) 50% extract from fully polymerized material.

The cytotoxicity test results using the MTS assay are shown in Figures 3 [Fig fig4] [Fig fig5] [Fig fig6] [Fig fig7]–8. In this study, the toxic effects on hNOF and IHOK were determined for five provisional resin materials in three different states of polymerization: the initial, intermediate, and final polymerized states. In the 50% extract coculture condition with hNOF, cell viability was less than 70% in all tested groups except JE3, RL2, RL3, LS1, LS2, and LS3 (Figure 3). Compared to the polymerized product, SN1, TR1, TR2, JE1, JE2, and RL1 showed significantly lower cell viability (Figure 3, *P* < 0.05).

In the 25% extract incubation of the hNOF, less than 70% cell viability was shown in SN1, TR1, and TR2, which were all from the chemically activated provisional resins (Figure 4). SN1, TR1, TR2, JE1, JE2, RL1, RL2, and LS1 had significantly lower cell viability compared to extracts from polymerizing materials (Figure 4, *P* < 0.05).

The results of the cytotoxicity tests were confirmed by confocal microscopic images of live and dead cells obtained after incubation in 50% extract in the supplemented medium. The results are shown in Figure 5, in which live cells are green and dead cells are red. A significant number of dead cells (red) but few live cells (green) appeared in either the initial or intermediate polymerizing state of SN, TR, JE, and RL, while similar numbers of viable cells (green) appeared in both LS specimens compared with the control.

The cytotoxicity test results using the MTS assay with IHOK are shown in Figures 6 [Fig fig7]–8. In the 50% extract coculture condition with IHOK, cell viability was less than 70% in all tested groups except JE3, RL1, RL2, RL3, LS1, LS2, and LS3 (Figure 6). Compared to the polymerized product, SN1, SN2, TR1, JE1, JE2, RL1, LS1, and LS2 showed significantly lower cell viability (Figure 6, *P* < 0.05).

In the 25% extract incubation of the IHOK, less than 70% cell viability was shown in SN1, SN2, SN3, TR1, TR2, and TR3, which were all from the chemically activated provisional resin (Figure 7). SN1, SN2, TR1, TR2, JE1, JE2, and LS1 had significantly lower cell viability compared to the extracts from the polymerizing material (Figure 7, *P* < 0.05).

The results of the cytotoxicity tests using 50% extract were confirmed by confocal live and dead microscopic images (Figure 8). A significant number of dead cells (red) but few live cells (green) appeared in the initial or intermediate polymerizing state of SN, TR, and JE, while similar numbers of viable cells (green) appeared in both RL and LS compared with the control specimens.

## 4. Discussion

Periodontal soft tissue management during treatment is important for fixed prosthodontic or complex implant restorations for maintaining aesthetically pleasing and healthy periodontal soft tissue. Before a permanent prosthodontic tooth is applied on a prepared tooth or implant abutment, an interim restoration using provisional resin materials is usually performed to enhance the aesthetic outcomes of periodontal tissue by restoring the gingival contour. During the fabrication process or restoration period, provisional resin materials can adversely affect periodontal tissue by extract or direct contact, which induces periodontal tissue management failure.

The mechanical properties of provisional resin materials are important for resisting severe biting force. If mechanical properties, including compressive strength, are lower, the material will break from biting force and damage the periodontal tissue. It is known that bis-acryl and UDMA, which are basic components of light-curable provisional resins, have better mechanical properties than do monomers of chemically activated provisional resins (MMA and EMA), which has been confirmed by the compressive strength results in this study. Therefore, from a mechanical point of view, it is better to use light-curable provisional resin materials for periodontal tissue management.

Cytotoxicity testing is a basic step in evaluating the biocompatibility of dental materials, including provisional resin materials, because it is a simple, economical, easily quantified, and clinically meaningful experiment that mimics the initial biological reaction in cell levels when dental materials contact dental tissue [22]. This study performed cytotoxicity testing using extracts from five types of provisional resin materials, including three chemically activated and two light-activated materials. Three different conditions were used for chemically activated materials to start extraction in distilled water: the initial polymerizing state (extraction starts from 1/4 of the setting time); the intermediate polymerizing state (extraction starts from 1/2 of the setting time); and the final “already set” stage (extraction starts from the setting time). For light-curable provisional resin materials, unpolymerized, partially polymerized by a short light-curing time (10 s), or fully polymerized by a long light-curing time (20 s) specimens were used for extraction. In terms of the dual curing system product (LS), chemical curing for 1 or 4 minutes or 4-minute chemical curing followed by 20 s of light curing was performed. The reason for using various extracting conditions is to mimic the conditions under which these materials are applied clinically around the periodontal soft tissue. Setting (or polymerizing) accelerates leaching or dissolved substances from the dental materials, which adversely affects the surrounding periodontal tissues. A low degree of resin polymerization tends to result in increased cytotoxicity due to an increase of released toxic components, particularly monomers, in all polymerizing products [24, 25].

Human oral mucosa consists of human oral keratinocytes and gingival fibroblasts, which play a major role in the healing and regeneration of periodontal tissue. When the above-mentioned cells are adversely affected by toxic chemicals or extracts, they lose their potency to maintain a healthy state and thereby induce inflammation or necrosis. According to the cytotoxicity test that used two types of oral cells, chemically active products have greater cytotoxicity than do light-curable products under all conditions (initial, intermediate, and “already set” stages), indicating that light-curable provisional resin materials are suitable choice for better periodontal tissue management outcomes. The monomers or eluates released from chemically activated acrylic resins might be toxic to various types of oral cells, such as keratinocytes, gingival fibroblasts, and dental pulp cells [26–28]. For most products, cytotoxicity from polymerizing chemically activated provisional resin materials was significantly higher than from fully polymerized materials [8]. To our knowledge, this study is the first to examine the cytotoxicity of chemically activated provisional resin materials against oral mucosa cells while they are polymerizing, and we found that these materials were severely cytotoxic to both oral keratinocytes and gingival fibroblasts.

Recent studies have reported that the substances that induce cytotoxic effects of provisional resin materials are mostly resin monomers, such as MMA, EMA, UDMA, and bis-acryl [16, 27]. These resin monomers can be released into the oral cavity and interfere with intracellular metabolic enzymes as well as with the production of fundamental proteins for regenerating periodontal tissue or maintaining its homeostasis [29, 30]. Among the materials we tested, EMA, MMA, UDMA, and bis-acryl were able to be involved in extraction and induced cytotoxicity. Along with previous cytotoxicity tests using various acrylic resin monomers, SN and TR polymerized from EMA were more cytotoxic than was JE polymerized from MMA [31]. In addition, LS and RL polymerized from bis-acryl and UDMA, respectively, showed lower levels of cytotoxicity than did chemically activated materials, primarily because of the hydrophobic characteristics of bis-acryl and UDMA. These different cytotoxic effects of monomers on oral cells can be explained by differences in lipophilicity because cell membrane lipids have the probability of being solubilized by the monomers, and cell membrane integrity can be damaged when monomers merge with the surface of the membrane's lipid bilayers [31, 32]. According to a previous study, MMA and EMA consist of traditional “chemically activated provisional resins” and belong to more lipophilic monomers than do bis-acryl and UDMA, which leads to a lack of biocompatibility as well as time-efficient characteristics, marginal adaptation, mechanical properties, and aesthetic performance [33, 34]. Therefore, to enhance the above-mentioned drawbacks, light-curable resins with bis-acryl or UDMA were introduced into provisional resin materials. Further quantitative and qualitative analyses using chromatography are needed to determine the major cause of cytotoxicity.

## 5. Conclusions

Our three null hypotheses were rejected. In terms of mechanical properties using compressive testing, chemically activated provisional resin materials had compromised properties compared to light-activated materials. In terms of cytotoxicity test, the light-activated products showed less adverse effects on the periodontal soft tissue cells in any polymerization stage compared to the chemically activated products. In addition, chemically activated products had significantly greater adverse effects during the “polymerizing” phase compared to those that were “already set.” In conclusion, the weakness of compressive strength and the possibility of cytotoxicity from chemically activated provisional resin materials, particularly during polymerization, should be taken into account when provisional resin materials are to be used around periodontal soft tissue after implant surgery and prosthodontic rehabilitation to avoid possible adverse effects from released toxic components on periodontal soft tissue.

## Figures and Tables

**Table 1:** Materials tested in this study.

Product	Code	Manufacturer	Lot number	Composition	Polymerizing type
Snap	SN	Parkell Inc., USA	01707	Polyethyl methacrylate (PEMA)	Chemical
Trim II	TR	Bosworth, USA	1409-442	Polyethyl methacrylate (PEMA)	Chemical
Jet	JE	Lang Dental, USA	40142	Polymethyl methacrylate (PMMA)	Chemical
Revotek LC	RL	GC America Inc., USA	1409191	Urethane dimethacrylate (UDMA)	Light
Luxatemp Solar	LS	DMG, Germany	726443	Bis-acryl composites	Light (dual cure)

**Table 2:** Experimental conditions of provisional resin materials.

Materials	Extraction starting time after the start of mixing			Polymerizing method
	Initial	Intermediate	After polymerization	
Snap	2 min, 30 sec (SN1)	5 min (SN2)	10 min (SN3)	Chemical
Trim Plus®	2 min (TR1)	4 min (TR2)	8 min (TR3)	Chemical
Jet	2 min, 30 sec (JE1)	5 min (JE2)	10 min (JE3)	Chemical
Revotek LC	Unpolymerized (RL1)	10 s (RL2)	20 s (RL3)	Light
Luxatemp Solar	1 min (LS1)	4 min (LS2)	4 min, curing 20 s (LS3)	Dual cure
